# Evaluating the Association between Assisted Conception and the Severity of Preeclampsia

**DOI:** 10.5402/2011/928592

**Published:** 2011-10-30

**Authors:** Kathryn C. Calhoun, Kurt T. Barnhart, Michal A. Elovitz, Sindhu K. Srinivas

**Affiliations:** ^1^Department of Obstetrics and Gynecology, University of North Carolina at Chapel Hill, 101 Manning Drive, Chapel Hill, NC 27599-7570, USA; ^2^Department of Obstetrics and Gynecology, The Hospital of the University of Pennsylvania, 3400 Spruce Street, Philadelphia, PA 19104, USA

## Abstract

*Objective*. To investigate the association between assisted conceptions and preeclampsia (PEC), including assessment of severity of disease. *Methods*. In a prospective case control study, cases were selected from women with preeclampsia and controls from women without preeclampsia. Exposure was defined as assisted conception with intrauterine insemination or in vitro fertilization (IUI or IVF). We assessed the association between exposure and outcome, using Chi square or Fisher's exact tests. Stratified analyses and multivariable logistic regression were used to control for confounders. *Results*. Preeclampsia was associated with assisted conception after controlling for age and race (AOR 2.2, [1.03–4.72]). All women with preeclampsia who had assisted conceptions demonstrated severe disease and were more likely to have abnormal lab values: AST >45 (AOR = 6.01 [1.63–22.21] *P* = 0.007), creatinine ≥1 (AOR 2.92 [0.82–10.4], *P* = 0.09) or platelets <100 (AOR 5.74 [1.00–32.76] *P* = 0.049), after adjusting for race, age, and multiple gestations. *Conclusion*. Assisted conceptions are associated with a more severe preeclamptic phenotype.

## 1. Introduction

Infertility affects approximately 12% of the reproductive age population, and in vitro fertilization (IVF) is employed for approximately 1% of live births in the United States [1]. IVF has been associated with subsequent maternal and fetal morbidity, including preeclampsia [2]. 

Preeclampsia affects 5–8% of all pregnancies, contributes to fetal morbidity, and is recognized as a leading cause of maternal mortality [3]. While the exact etiology of the disease remains poorly understood, it is theorized that preeclampsia stems from events related to placentation [4–6]. Despite this small window of time for a seemingly uniform early pregnancy insult, preeclampsia has a large spectrum of disease presentation, from simple hypertension to end-organ damage involving multiple organ systems.

Because assisted conceptions involve more exogenous manipulation of the embryo and/or uterine milieu, we hypothesized that we would find a more severe phenotype of preeclamptic disease in these pregnancies. While previous studies have reported associations between pregnancy-related hypertensive disease and IVF in both singleton pregnancies and multiple gestations, no study has examined the link between assisted conceptions and disease phenotype, specifically the severity of disease [2, 7–13]. 

The objectives of this study were to further explore the association between assisted conceptions and preeclampsia and to specifically examine the association between mode of conception, severity of preeclampsia, and end-organ involvement.

## 2. Materials and Methods

We performed a planned secondary analysis of patients enrolled in a case-control study, Preeclampsia: Mechanisms and Consequences between March 2005 and August 2007 at the Hospital of the University of Pennsylvania. 

Institutional Review Board approval was obtained prior to enrollment. Cases were defined as women with gestational hypertension or preeclampsia. Controls were defined as women presenting for delivery at term (≥37 weeks) without gestational hypertension or preeclampsia. All women admitted to Labor and Delivery with preeclampsia were eligible for enrollment and invited to participate in the overall study. Cases were identified based on maternal criteria for preeclampsia. Controls were prospectively enrolled from all women presenting for delivery at term (≥37 weeks) for scheduled induction of labor, scheduled cesarean section, spontaneous rupture of membranes, or term labor. There were no exclusion criteria for either cases or controls in the overall study. Cases and controls were frequency matched for race (African American versus other).

Diagnostic criteria for preeclampsia were determined per ACOG guidelines, and disease classification was determined by the primary investigators (SKS and MAE) and not by the physician caring for the patient [14]. Mild preeclampsia included the diagnosis of gestational hypertension and was defined as elevated blood pressure (140/90 mmHg or greater on two measurements that were obtained six or more hours apart, or 160/105 mmHg or greater at time of admission) with proteinuria ≤+1 on urine dip. Severe preeclampsia was defined as blood pressure 160/105 mmHg with greater than +1 on urine dip or ≥140/90 mmHg on two occasions 6 hours apart and any one of the following: platelets <120,000/mL, aspartate aminotransferase (AST) >45 U/L, alanine aminotransferase (ALT) >60 U/L, and/or creatinine ≥1.0. Severe disease also included women diagnosed with HELLP (Hemolysis, Elevated Liver Enzymes Low Platelets) syndrome or eclampsia, women who required intravenous antihypertensive medications prior to delivery (as a surrogate for blood pressure values persistently in the severe range), or iatrogenic preterm delivery (<37 weeks) due to the severity of their disease. Additional descriptions of this study are provided elsewhere [15, 16]. 

Trained research nurses collected information on height, race, ethnicity, conception history (spontaneous or assisted), and history of chronic hypertension (CHTN) by patient interview at the time of enrollment. Other history including obstetric, demographic, prenatal, delivery, and neonatal information was collected from prenatal and hospital chart abstraction by trained research nurse abstractors. A separate individual performed data entry. Intrauterine growth restriction was defined using actual birth weight and the Alexander growth curve to determine percentile fetal growth for gestational age [17].

In our study, exposure was defined as assisted conception and further subdivided into intrauterine insemination (IUI) or in vitro fertilization (IVF). The IUI group included use of clomiphene citrate and injectable gonadotropins. The IVF group also included all conceptions with or without intracytoplasmic sperm injection (ICSI).

Chi square analysis or Fisher's exact tests were used to compare categorical variables, and Student's t tests were used for normally distributed continuous variables. Confounding variables were selected based on biologically plausible associations, prior literature reports, or statistical significance. Multivariable logistic regression was used to control for the following confounders: maternal age, maternal race, and multiple gestation. A *P* value of less than 0.05 was considered statistically significant.

We performed an initial analysis to confirm the previously reported association between the use of assisted conception and preeclampsia [2]. We subsequently performed analyses restricted to only women with preeclampsia (cases only). The purpose of these subsequent analyses was to evaluate the association between assisted conception and severity of disease (phenotype) among women with preeclampsia. The comparison of maternal and pregnancy characteristics and severity of preeclampsia between assisted and unassisted pregnancies was performed using Chi square and Fisher's exact tests. Multivariable logistic regression was used to control for confounders. Finally, analyses were also performed restricted to those who conceived with IVF compared to those with an unassisted conception.

## 3. Results

A total of 1031 patients were enrolled in the overall study. 440 women were diagnosed with preeclampsia, and 591 women were controls. Within the cases, 16 women (3.6%) had assisted conceptions (4 IUI, 12 IVF), and 424 women had unassisted pregnancies. Within the control population, 16 women (2.7%) had assisted conceptions (11 IUI, 5 IVF), and 575 women conceived without assistance. 

Table 1 shows a demographic comparison of the cases versus controls. Cases were more likely to be African American and primiparous. Cases were also more likely to be carrying multiple gestations and to have a history of chronic hypertension (CHTN) and a previous history of preeclampsia.

An unadjusted analysis demonstrated that women with preeclampsia (cases) had a 1.4 times higher odds (CI 0.63–2.93, *P* = 0.4) of having an assisted conception than women without preeclampsia. However, after controlling for age and race, women with preeclampsia had a two-times higher odds of having an assisted conception than women without preeclampsia (AOR 2.2, [1.03–4.72], *P* = 0.042). The association was stronger when the adjusted analysis (adjusted for age, race, and multiple gestation) was restricted to conception after IVF (AOR 5.3, [1.74–15.89], *P* = 0.003). No association was noted between conception with IUI and preeclampsia (AOR 0.83, [0.25–2.72], *P* = 0.76) (Table 2). 

We next performed analyses restricted to only women with preeclampsia (cases only).  Table 3 shows a demographic comparison of preeclamptic women that conceived with and without assistance. Women with preeclampsia with assisted conceptions were more likely to be older and non-African American, and to be carrying multiple gestations than those women with preeclampsia and unassisted conceptions. A disease phenotype comparison between the two groups revealed severe preeclampsia in all cases with assisted conceptions. This severity manifested as proteinuria and laboratory findings consistent with the end-organ damage associated with HELLP syndrome rather than as significant hypertension (Table 4). After controlling for age, race, and multiple gestations, there was a significant association between assisted conception and laboratory findings consistent with end-organ damage: elevated AST (AOR 6.01 [1.63–22.21], *P* = 0.07), platelets less than 100,000 (AOR 5.74 [1.00–32.76], *P* = 0.049), and elevated creatinine (AOR 2.92 [0.82–10.4], *P* = 0.09). 

A subsequent analysis further restricted to preeclamptic women who conceived with IVF (excluding IUI) also demonstrated evidence of the same end-organ damage: elevated AST (AOR 4.9 [1.00–24.31], *P* = 0.05), platelets less than 100,000 (AOR 18.2 [1.6–21.03], *P* = 0.02), and elevated creatinine (AOR 8.0 [1.45–44.21], *P* = 0.02), compared to women with preeclampsia with unassisted conceptions. 

## 4. Discussion

In our study, we were able to demonstrate a novel difference in the severity of the phenotype of preeclampsia based on mode of conception. Women with assisted conceptions, particularly those who had employed IVF, were significantly more likely to manifest a severe disease characterized by laboratory findings consistent with end-organ damage. Abnormalities in transaminases, platelets, and creatinine suggest liver, endothelial, and renal damage, respectively, which can have implications for acute and chronic maternal morbidity and neonatal morbidity. Recent population-based retrospective cohort studies have illustrated this potential for ongoing morbidity, suggesting that pregnancy-related hypertensive disorders are associated with maternal risk of early-onset cardiovascular disease and death from cardiovascular causes [3, 18].

Infertility affects a growing subset of the U.S. population, and accordingly, assisted conceptions will continue to account for a larger proportion of births. Studies focusing on maternal and fetal outcomes following assisted conception have identified pregnancy-related hypertensive disorders among the potential associated adverse outcomes. A 2004 meta-analysis by Jackson et al. comparing 12,283 IVF singleton births to 1.9 million unassisted singleton births detected higher rates of morbidity in the IVF group including preeclampsia [8]. Shevell et al. compared spontaneous pregnancies (*n* = 36, 062) to pregnancies conceived with ovulation induction (*n* = 1222) and IVF (*n* = 554) and found that IVF was shown to be associated with gestational hypertension (OR 1.6 [1.0–2.5], *P* = 0.036), preeclampsia (OR 2.7 [1.7–4.4], *P* < 0.001), placental abruption (OR 2.4 [1.1–5.2], *P* = 0.03), and placenta previa (OR 6.0 [3.4–10.7], *P* < 0.001) [2]. The etiology of these associations is unknown, but, with more women seeking fertility therapies, it is essential that we expand our knowledge, as gestational hypertensive disorders have implications for significant acute and long-term morbidity and mortality.

The strengths of our study are that it includes extensive measurements of prenatal, intrapartum, and postnatal data. Both the exposure and the outcome were prospectively collected and specifically, information regarding the mode of conception was collected at the time of enrollment by patient interview. Further, diagnosis and severity of hypertensive disease were decided from standardized, objective, prespecified criteria rather than by physician report. Additionally, the extent of our disease severity data and the number of women with preeclampsia in our case control study allowed us to evaluate severity of preeclampsia associated with assisted conceptions in our population.

An additional strength was the use of logistic regression to control for potential confounding factors such as differences in age, race, and multiple gestations between our groups. Despite the inherent reduction in power with a multivariable analysis, we were able to demonstrate for the first time that, among women with preeclampsia, those with assisted conceptions manifested a more severe disease phenotype.

There are also some limitations to our study. The number of pregnancies conceived with medical assistance, though representative of our population, is small. Further, information regarding the details of the assisted conceptions (dose of medication or culture conditions used in IVF) and the etiology of the patients' infertility were unavailable. Additionally, while some may have excluded women with chronic hypertension and a history of preeclampsia, we believe that the inclusion of these patients is important, makes our findings more generalizable, and should not impact our within-case analyses evaluating disease severity. Lastly, in performing a case control study, the control population is subject to scrutiny. We did not match controls by gestational age. However, the association between IVF and preeclampsia is not gestational age dependent and has been demonstrated in previous work [2, 8]. Further, our analyses evaluating the disease severity was restricted to women with preeclampsia and does not involve the control population.

Possible mechanisms for the association between assisted conception and preeclampsia include defects or deficiencies in implantation or placentation, and/or problems with the uterine milieu or in vitro culture conditions. Reports of increased rates of preeclampsia and gestational diabetes, together with abruption and previa in pregnancies conceived with IVF, certainly favor a placental origin for these adverse outcomes. During implantation, fetal trophoblasts invade the decidua to eventually access the maternal vasculature. Truncated or flawed vascular invasion may result in placental insufficiency, and an inability to respond to the demands of a growing pregnancy may trigger third-trimester pathologies such as preeclampsia [4]. When assisted conceptions were divided into IVF and IUI, IVF was associated with a significantly increased risk of preeclampsia whereas IUI was not. The increased manipulation of embryo and uterine milieu in IVF cycles may affect implantation and possibly predispose to more severe third-trimester pathologies. Invasion of maternal vasculature may be quantitatively or qualitatively different from an in vivo conception, thus predisposing to preeclampsia by disruption of placental events by the pathways theorized above. 

## 5. Conclusion

While the majority of children conceived with medical assistance are born healthy after an uncomplicated pregnancy, this paper presents novel information regarding the association between assisted conception and altered maternal and perinatal outcomes, in that a more severe phenotype, with suggestion of end-organ damage, was seen in assisted conceptions. 

Future research should examine the impact of fertility treatments on placentation and third-trimester morbidity. In addition, women seeking fertility treatments should be counseled that these risks exist and that increased surveillance in the third trimester may be warranted.

## Figures and Tables

**Table 1:** Demographics: Preeclamptic cases versus non-preeclamptic controls.

Variable	Preeclampsia % (*N* = 440)	Control % (*N* = 591)	*P* value
Mean age	26.8 +/− 6.9	27.2 +/− 6.6	0.42
Race (African American)	84.8 (373)	74.5 (440)	0.0001
Mean BMI at screening	29.9 +/− 8	28.9 +/− 7.6	0.06
Primiparous	33.4 (147)	22.7 (134)	0.0002
Mean gest age at delivery	36.1 +/− 3.7	39.2 +/− 1.2	<0.001
Twin Pregnancy	5.2 (23)	0 (0)	<0.0001
Pregestational diabetes	4.1 (18)	2.2 (13)	0.12
Gestational diabetes	3.9 (17)	2.2 (13)	0.17
Chronic hypertension	15.5 (68)	5.3 (31)	0.0001
History of preeclampsia	20.9 (92)	7.6 (45)	0.0001
History of preterm preeclampsia	9.8 (43)	2.0 (12)	0.0001
Tobacco use	12.1 (53)	10.3 (61)	0.44

**Table 2:** Assisted conception: preeclamptic cases versus non-preeclamptic controls^†^.

Type of treatment	Unadjusted OR	95% CI	Adjusted OR	95% CI	*P* value
All assisted^‡^ (IVF + IUI)	1.4	0.63–2.93	2.2	1.03–4.72	0.042
IVF^§^	3.3	1.1–11.9	5.3	1.74–15.89	0.003
IUI^¶^	0.49	0.11–1.7	0.83	0.25–2.72	0.76

^†^Adjusted for age and race.

^‡^Assisted pregnancies included use of clomiphene citrate, gonadotropins, IUI, IVF, ICSI, and donor egg.

^§^IVF pregnancies employed in vitro fertilization for conception.

^¶^IUI group included use of clomiphene citrate and gonadotropins. IVF was not used in this group.

**Table 3:** Demographics within preeclamptics.

Variable	Assisted^†^ % (*N* = 16)	Unassisted^‡^ % (*N* = 424)	*P* value
Mean age	37.3 +/− 5	26.4 +/− 6.7	<0.001
Race (African American)	18.8 (3)	87.3 (370)	<0.0001
Mean BMI at screening	27.8 +/− 6.8	30 +/− 8	0.31
Primiparous	75 (12)	50.5 (214)	0.095
Gestational age at delivery	33.8 +/− 4.2	36.1 +/− 3.7	0.01
Twin pregnancy	56.3 (9)	3.1 (13)	<0.0001
Pregestational diabetes	0	4.3 (18)	0.84
Gestational diabetes	6.3 (1)	3.8 (16)	0.88
Chronic hypertension	6.3 (1)	15.8 (67)	0.49
History of preeclampsia	18.8 (3)	21.0 (89)	0.93
History of preterm preeclampsia	6.3 (1)	11.8 (50)	0.78
Tobacco use	0	12.5 (53)	0.26

^†^Assisted pregnancies included use of clomiphene citrate, gonadotropins, IUI, IVF, ICSI, and donor egg,

^‡^Women with unassisted pregnancies did not report use of clomiphene citrate, gonadotropins, IUI, IVF, ICSI, or donor egg for conception.

**Table 4:** Analysis of preeclamptic disease severity: assisted versus unassisted conceptions.

Variable	Assisted^†^ % (*N* = 16)	Unassisted^‡^ % (*N* = 424)	*P* value
Severe disease	100 (16)	60.9 (258)	0.0036
Systolic BP >160	56.3 (9)	78.8 (334)	0.068
Diastolic BP >105	37.5 (6)	34.7 (147)	0.97
IV BP medication	25 (4)	25.2 (107)	0.79
Proteinuria ≥2+	75 (12)	35.4 (150)	0.0031
Creatinine ≥1.0	50 (8)	18.6 (79)	0.0056
AST ≥45	62.5 (10)	20.3 (86)	0.0002
ALT ≥60	31.3 (5)	11.3 (48)	0.0441
Platelets <100	25 (4)	6.4 (27)	0.0182
IUGR <10%	50 (8)	27.4 (116)	0.09

^†^Assisted pregnancies included use of clomiphene citrate, gonadotropins, IUI, IVF, ICSI, and donor egg,

^‡^Women with unassisted pregnancies did not report use of clomiphene citrate, gonadotropins, IUI, IVF, ICSI, or donor egg for conception.
